# Supplementary material for: Targeting of Phospholipase D1 Ameliorates Collagen-Induced Arthritis via Modulation of Treg and Th17 Cell Imbalance and Suppression of Osteoclastogenesis
Source: Int J Mol Sci. 2020 May 2;21(9):3230. doi: 10.3390/ijms21093230 (PMC7247592; doi:10.3390/ijms21093230)
Supplement: Supplementary file 1 [file ijms-21-03230-s001.pdf]

SUPPLEMENTARY MATERIALS AND METHODS

Immunohistochemistry

The hind legs of the mice were removed, fixed with 4% paraformaldehyde in PBS, decalcified for 10 days with 10% EDTA, embedded in paraffin, and sectioned at 7 μm thickness. After the sections were deparaffinized, rehydrated and washed, the sections were then antigen retrieved with 10 mM citrate buffer (pH 6.0) and incubated with 0.3% hydrogen peroxidase for 20 min to block endogenous peroxidase activity, followed by processing with 10% goat serum for 30 min to block non-specific ligations. The sections were then treated with primary antibodies, including anti-TNF-α (1:200, Abcam, Cambridge, MA, USA), anti-IL-1β (1:100, Santa Cruz Biotechnology, Santa Cruz, CA, USA), anti-IL-6 (1:100, Abcam), anti-IL-17 (1:100, Santa Cruz Biotechnology), anti-IFN-ɣ (1:200, Santa Cruz Biotechnology) and anti-MCP-1 (1:200, Abcam) overnight at 4˚C. After washing PBS three times, sections were incubated with biotinylated secondary antibody, and the signal was amplified with HRP-conjugated streptavidin using a Vectastain Elite ABC kit (Vector, Burlingame, CA) and visualized with diaminobensidine (DAB; Sigma-Aldrich).

Cytokine analysis in splenocytes

To determine cytokine levels *in vitro*, splenocytes (2×10^5 cells/mL) isolated from normal, vehicle or PLD1-inhibitor-treated CIA mouse, were stimulated with 50 μg/ml heat-denatured CII (T cell Proliferation Grade, Chondrex) and/or PLD1 inhibitor (5 μM) for 48 h in 24-well plates. Supernatants were collected from each well, and the levels of IL-17 were determined using ELISA kits (eBioscience, Hatfield, UK) according to the manufacturer's recommendations.

Coculture assays

Peripheral blood mononuclear cells (PBMC) from healthy donors were isolated by Ficoll-Paque^{TM} PLUS (GE Healthcare, Sweden) density-gradient centrifugation according to Institutional Ethics Review Committee of the Seoul National University (IRB No. H-0906-032-283, approved on 21 August 2009). RASF were prepared from synovial tissues of patients with RA who underwent knee joint replacement surgery or synovectomy according to the protocol approved by Institutional Ethics Review Committee of the Seoul National University Hospital (IRB No. H-0704-002-203, approved on 6 August 2007). RA was diagnosed according to the criteria of the American College of Rheumatology. Synoviocytes were microdisected from human RA synovium, minced, and digested with 1 mg/mL collagenase (Wortington Biochemical, Lakewood, NJ, USA) at 37˚C for 4 h. The cell suspension was cultured in Dulbecco's modified Eagle's medium (DMEM; WelGENE Inc., Daegu, South Korea) supplemented with 10% heat-inactivated fetal bovine serum (FBS; Gibco, Rockvile, MD, USA) and 1% antibiotics (penicillin/streptomycin) in a humidified 5% CO_2 atmosphere. Coculture was initiated by seeding RASF overnight in 24-well plates at density of 1×10^5 cells/well. The next day, anti-CD3/CD28 mAbs (BD Biosciences, San Diego, CA, 2 μg/ml each one)-activated PBMC (1×10^6 cells/well) were seeded and pretreated with PLD1 inhibitor (5 μM), treated with TNF-α (10 ng/mL) or LPS (100 ng/mL) for 36 h. The secreted cytokines were measured by ELISA.

Histopathology

The liver and spleen tissues were fixed with 4% paraformaldehyde in PBS for 18 h and then embedded in paraffin. Sections of 5 μm thickness were cut and stained with hematoxylin and eosin (H&E). H&E-stained tissue sections were examined, and inflammation was graded. A histological assessment was performed by a blinded observer, who scored the liver sections

using the following criteria: normal histology. ‘0’; minor hepatocellular death and inflammation, ‘1’; widely distributed patch necrosis and inflammation, ‘2’; complete disruption with panlobular necrosis and inflammation, ‘3’; and mortality, ‘4’. Spleen was scored for the enlargement of lymphocyte areas in red and white pulps (0, absent; 1, slight; 2, mild; 3, moderate; and 4, pronounced). Stained tissue sections were imaged using light microscope (Olympus, Tokyo, Japan) provided with digital camera with a high resolution.

SUPPLEMENTARY FIGURES

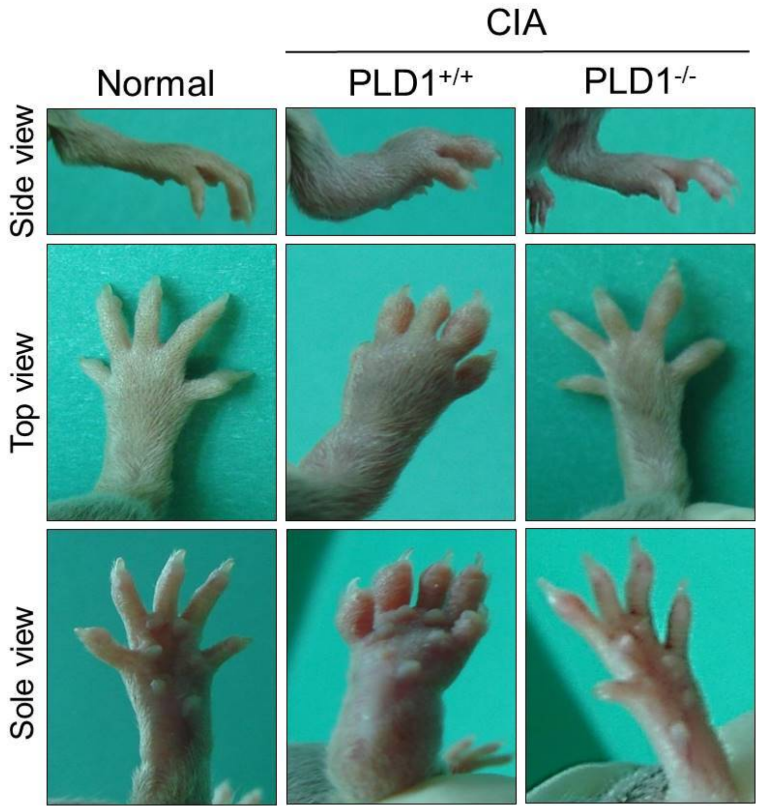

Figure S1. PLD1 deficiency ameliorates inflamed paws.

Representative photographs of showing the gross features images of hind paws on PLD1-ablated mice with CIA.

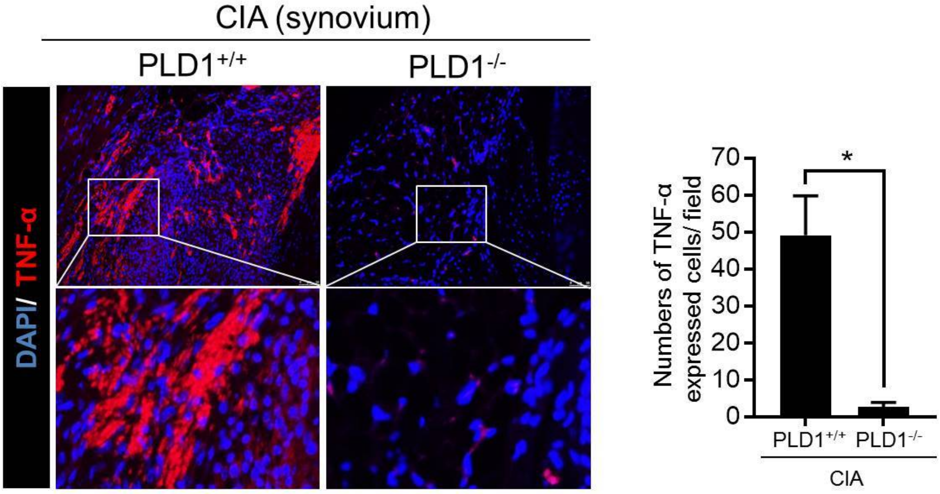

Figure S2. Immunofluorescence staining of TNF-α in the synovium tissues from CIA-challenged PLD1$^{+/+}$ and PLD1$^{-/-}$ mice.

The sections of knee joint from the indicated mice with CIA at day 42, were analyzed by immunofluorescence staining using anti-TNF-α antibody and DAPI. The number of TNF-α-expressed cells was quantified. Data are representative of at least three independent experiments and shown as the mean ± SEM. * $P < 0.01$

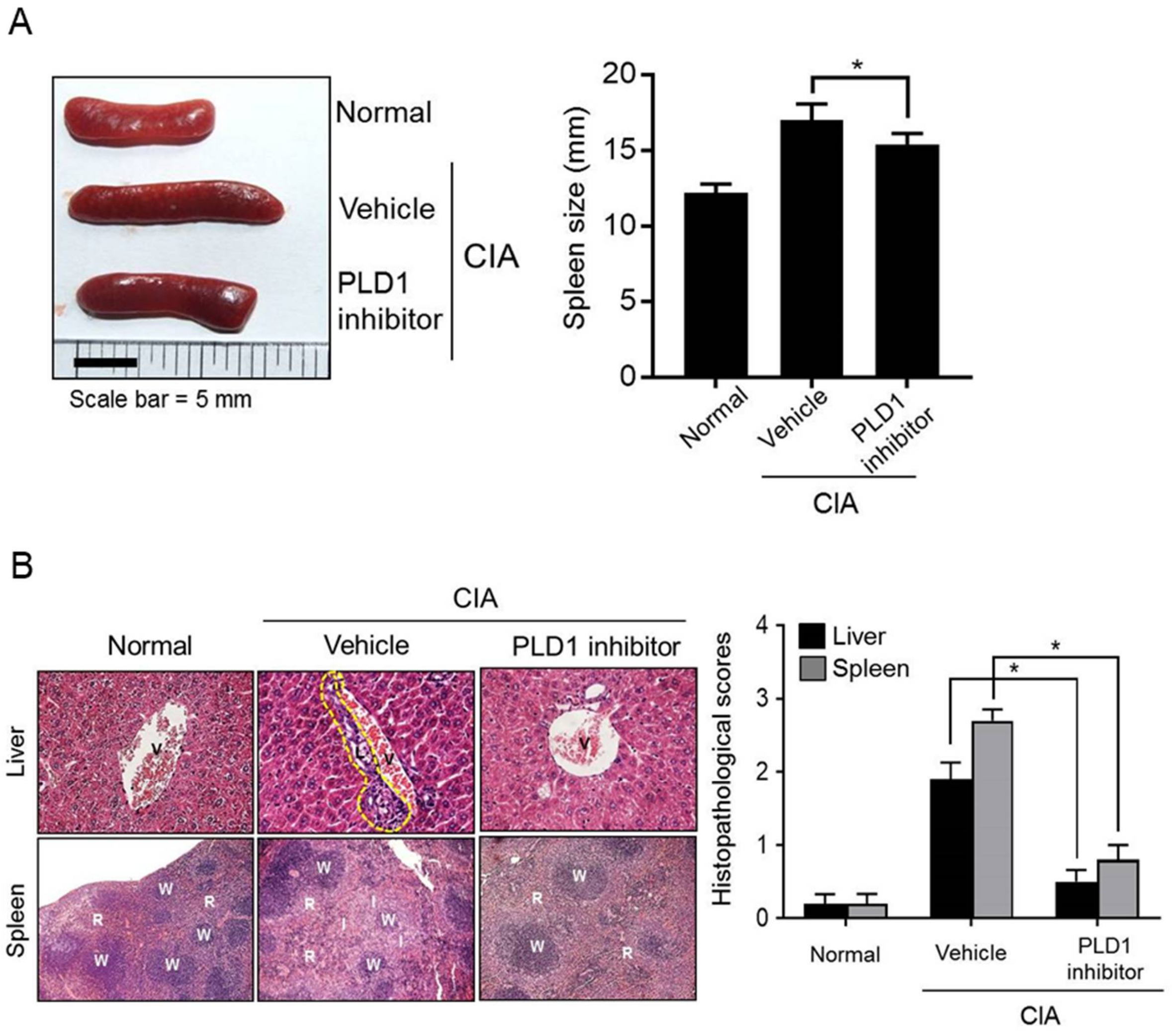

Figure S3. PLD1 inhibition reduces the size of spleen, inflammatory cells from mice with CIA. (A) Representative images of spleen from the indicated mice (n=15, at day 45) and quantification of the spleen size (B) Representative images of H&E-stained sections and histopathological scores of liver and spleen from the indicated mice at day 45. Data are representative of at least three independent experiments and shown as the mean ± SEM. $^{*}p < 0.05$. Lymphocyte infiltration is observed in the areas in the yellow dotted lines. V, Vein; L, lymphocytes; R, red pulp; W, white pulp; I, Inflammatory lesion.

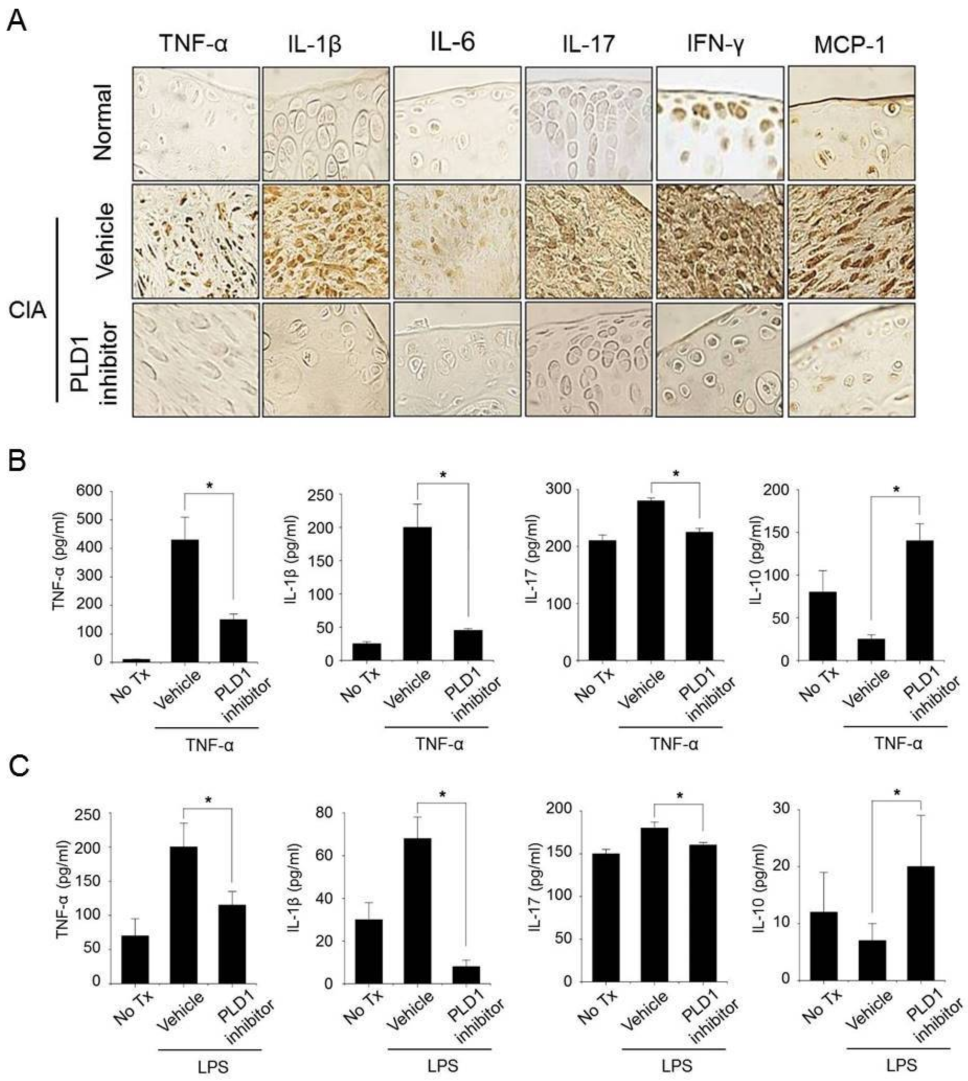

Figure S4. PLD1 inhibitor suppresses the expression of proinflammatory cytokines in CIA mice and RASF cocultured with PBMC.

(A) The knee joints from the indicated mice (n=6, at day 45) were analyzed by immunohistochemistry using antibodies to the indicated proinflammatory cytokines and chemokine. Data are representative of at least three independent experiments. (B, C) Human RASF (n=3) cocultured with activated human PBMC (n=3) were treated with PLD1 inhibitor (10 μM) and stimulated with TNF-α (10 ng/mL) or LPS (100 ng/mL) for 36 h. Production of proinflammatory cytokines in the supernatant was measured by ELISA. Data are representative of at least three independent experiments and shown as the mean ± SEM.

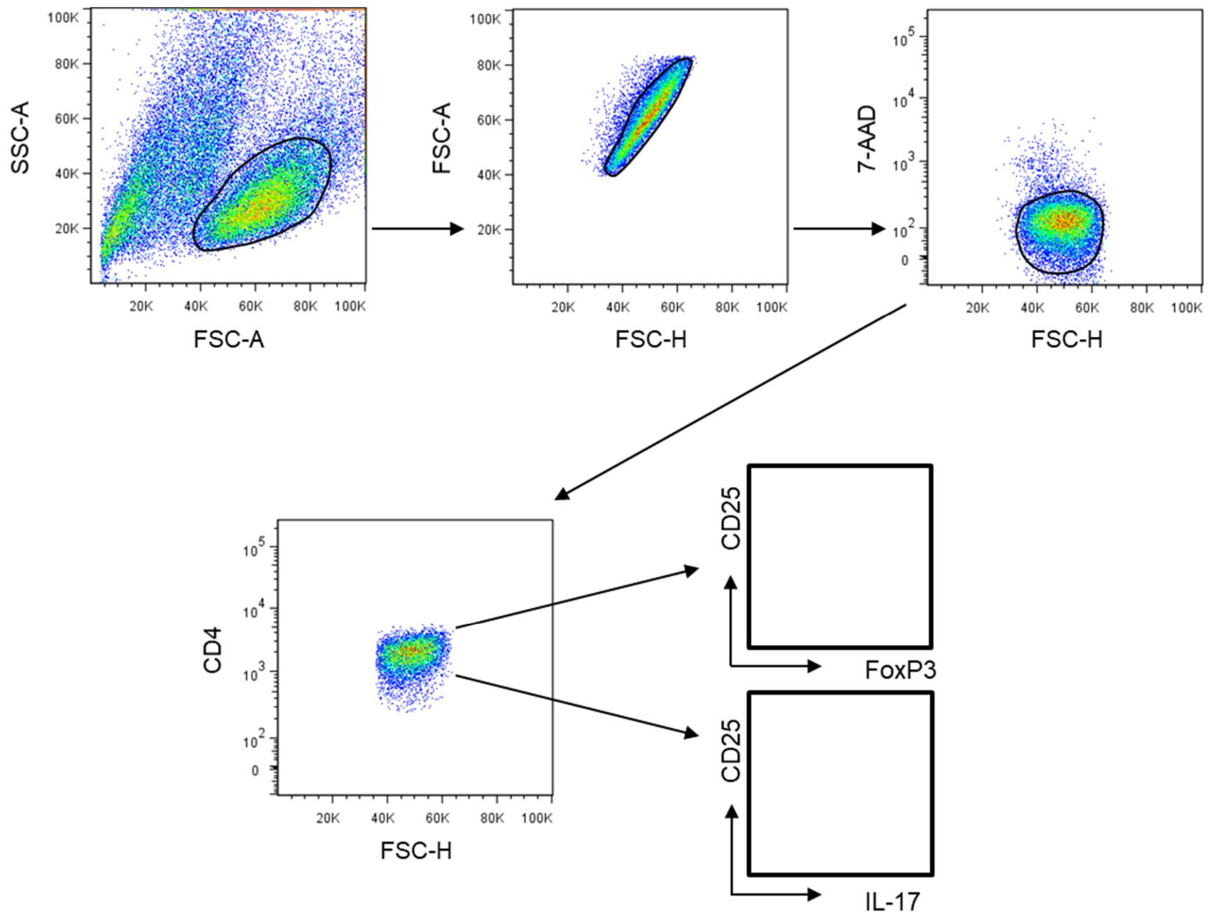

Figure S5. The flow cytometry gating strategies the staining of intracellular Foxp3 and IL-17. A gate based of forward scatter area and side scatter area is first established, then dead cells and doublets were eliminated. Next, $CD4^+$ T cells were gated, and then analyzed for $CD25^+Foxp3^+$ or $CD25^+IL\text{-}17^+$ cells.

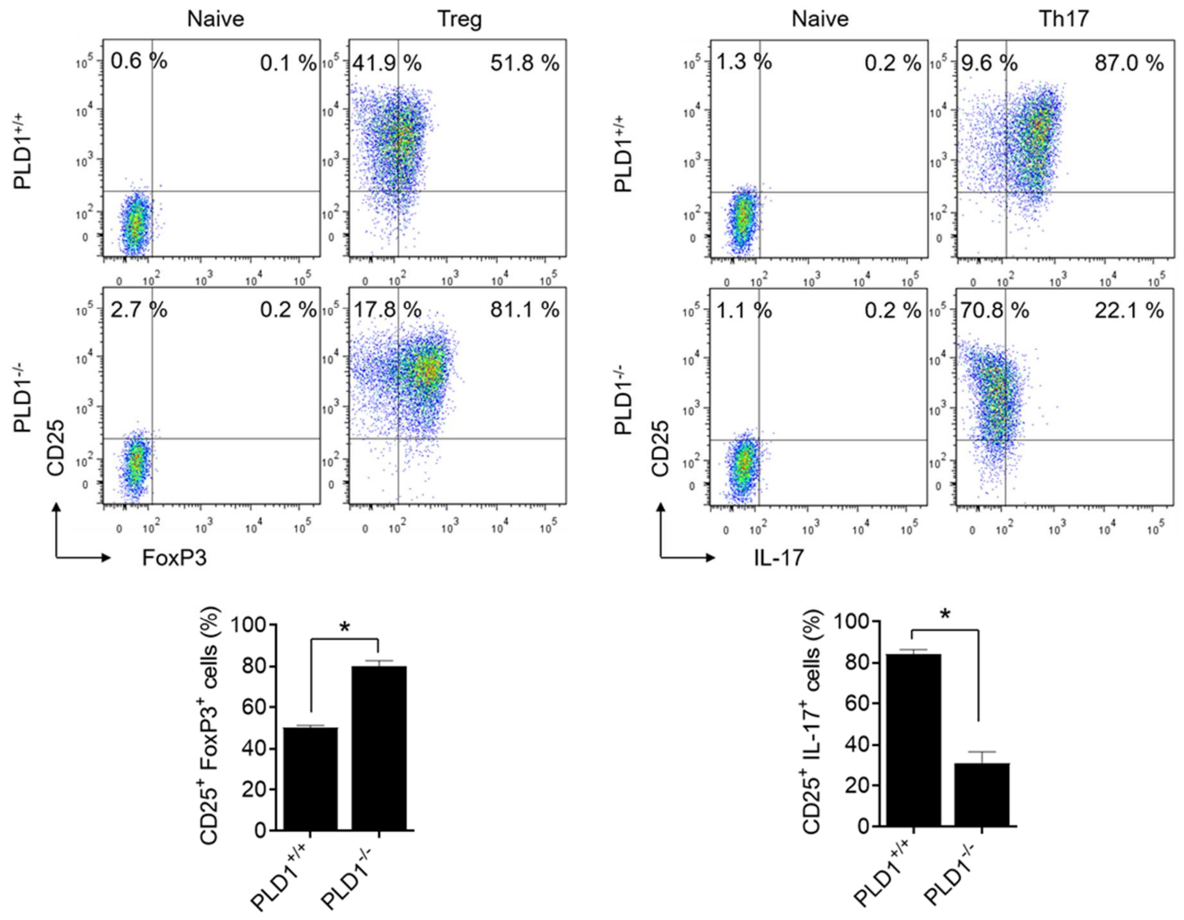

Figure S6. Effect of the targeting PLD1 on *in vitro* differentiation of Treg and Th17 cells. Naïve $CD4^+$ T cells isolated from the indicated mice spleens (n=6) were cultured under condition of Treg cells or Th17 cell differentiation. Representative flow cytometry profile and quantification for $CD25^+Foxp3^+$ Treg and $CD25^+IL\text{-}17^+$ Th17 cell populations.

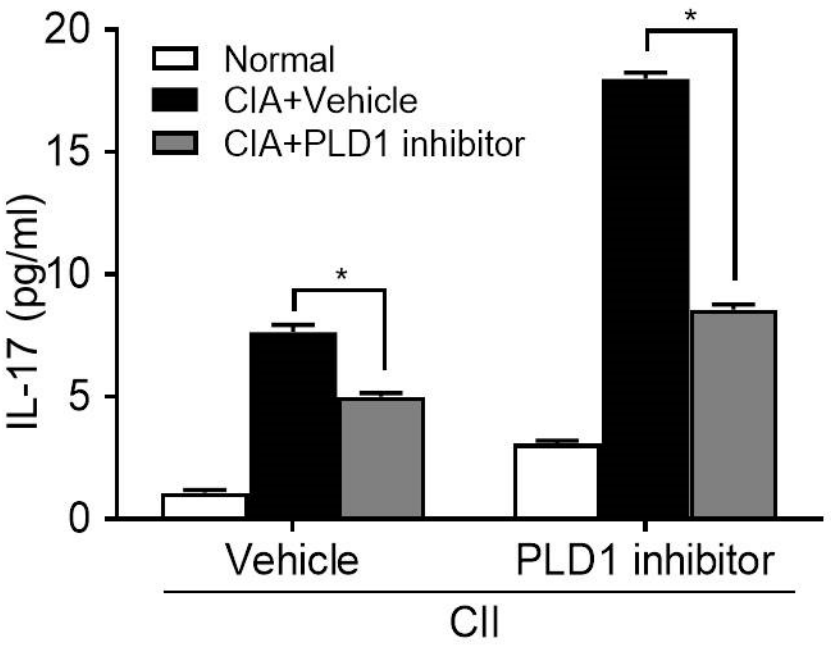

Figure S7. PLD1 inhibitor reduces production of IL-17 in splenocytes.

The splenocytes were isolated from the indicated mice at day 45 and stimulated with heat-denatured CII in the presence or absence of PLD1 inhibitor. The amount of IL-17 was measured from the supernatant by ELISA. Data are the mean ± SEM of three independent experiments. *$p < 0.05$

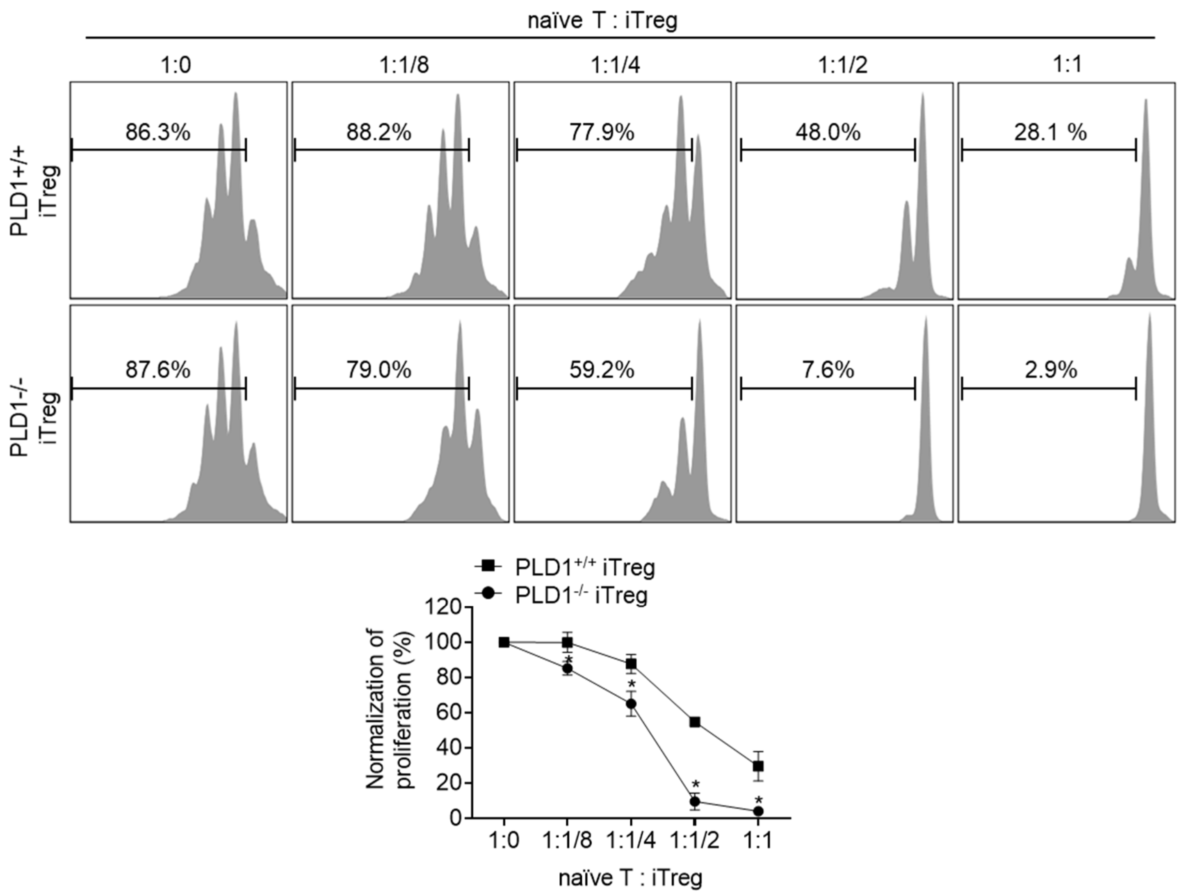

Figure S8. iTreg cells differentiated from PLD1-deficient T cells suppress proliferation of naïve T cells.

Fresh isolated naïve CD4$^+$ T cells, labeled with CFSE were cocultured in the presence of anti-mouse CD3ε and anti-mouse CD28 for 72 h with induced Treg cells differentiated from PLD1$^{+/+}$ and PLD1$^{-/-}$ T cells (n=6). Proliferation of naïve T cells was analyzed by flow cytometry for CFSE dilution

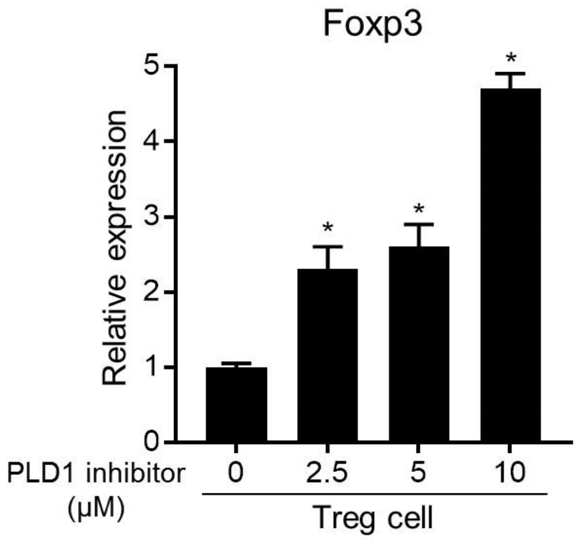

Figure S9. PLD1 inhibitor enhances the expression of Foxp3 during differentiation of Treg. During differentiation of Treg cells, the indicated concentration of PLD1 inhibitor was treated and expression of Foxp3 was analyzed by q-PCR. Data are shown as the mean ± SEM of three independent experiments. $*p < 0.05$
